# Oral health care challenges in individuals with severe mental illness: a qualitative meta-synthesis

**DOI:** 10.3389/froh.2025.1655450

**Published:** 2025-09-12

**Authors:** Tanzina Afroz, Joseph Beyene, Khaleda Zaheer, Mohamad Alameddine, Mohammad Hayatun Nabi, Mohammad Delwer Hossain Hawlader, Ahmed Hossain

**Affiliations:** 1 Department of Public Health, North South University, Dhaka, Bangladesh; 2 Department of Health Research Methods, Evidence, and Impact, McMaster University, Hamilton, ON, Canada; 3 Refugee Crisis Foundation, London, United Kingdom; 4 College of Health Sciences, University of Sharjah, Sharjah, United Arab Emirates

**Keywords:** mental health, oral health, dental health, barriers to oral health care, qualitative study

## Abstract

**Background:**

Individuals with severe mental illness (SMI) experience significantly higher rates of poor oral health, including dental caries, periodontal disease, and edentulism, compared to the general population. This meta-synthesis investigates the challenges faced by individuals with SMI in managing oral health and potential solutions.

**Methods:**

A comprehensive literature search (2010–2024) was conducted across PubMed, Web of Science, Scopus, and Google Scholar for any-language studies. The meta-synthesis involved systematic article selection, quality appraisal, and thematic data extraction/synthesis.

**Results:**

From 1,698 records, 101 full-text articles were reviewed; 11 met the inclusion criteria. Findings consistently demonstrate a high prevalence of poor oral health outcomes (caries, tooth loss, periodontal disease) among individuals with SMI, alongside significantly lower engagement in oral hygiene (e.g., toothbrushing) and dental care-seeking behaviours. Key barriers include financial constraints, dental anxiety, medication side effects (notably xerostomia), and low oral health awareness. Stigma and inadequate dental professional training in mental health further impede access. Proposed solutions emphasise integrating oral health education into psychiatric rehabilitation, enhancing communication between dental and mental health providers, and developing tailored support systems. Evidence suggests a bidirectional relationship between oral and mental health.

**Conclusion:**

This meta-synthesis confirms a stark oral health disparity for individuals with SMI, driven by suboptimal hygiene, medication effects, limited health literacy, and formidable access barriers compounded by financial hardship and stigma. Addressing this requires urgent, coordinated integration of mental and oral healthcare through co-located services, interdisciplinary collaboration, and tailored interventions. Future research must prioritise quantitative studies to elucidate causal pathways and long-term impacts, rigorously examining the roles of gender, geography, environment, and comorbidities. Bridging this divide is an essential public health imperative demanding systemic reform.

**Systematic Review Registration:**

https://www.crd.york.ac.uk/PROSPERO/view/CRD42024516535, identifier PROSPERO [CRD42024516535].

## Introduction

Mental illness significantly impacts oral health, creating a complex interplay between psychological and physical well-being. Individuals with mental disorders often experience poor oral health, which can exacerbate their overall health challenges (1, 2). People living with mental illnesses often face unique barriers to accessing and maintaining adequate oral health, resulting in disproportionately higher rates of oral diseases compared to the general population (3). These challenges are multifaceted, stemming from factors such as medication side effects, cognitive impairments, socioeconomic disparities, and stigmatization within healthcare systems (3, 4).

Mental illness encompasses a wide range of psychological conditions that affect how individuals think, feel, and behave, often leading to significant challenges in daily functioning and overall well-being. Globally, mental health disorders are highly prevalent, with conditions such as anxiety and depression affecting millions each year and contributing to substantial economic and social burdens (5, 6). For instance, it is estimated that mental illnesses account for 7.4% of disability-adjusted life years (DALYs) and 22.7% of global years lived with disability (YLDs), underscoring their role as a leading cause of disability worldwide (7). Furthermore, the prevalence of mental health disorders increased during the COVID-19 pandemic (8–10). Despite growing awareness and improved diagnostic efforts, mental health remains underreported in many regions, with systemic gaps in care and support.

An overlooked aspect of severe mental health illnesses (SMI) and their intersection with oral health. Individuals with SMI have a high risk of poor dental health due to factors such as self-neglect, adverse effects of psychotropic medications (e.g., xerostomia), substance use, and financial constraints (11–13). Poor oral health can exacerbate mental health conditions and vice versa, creating a bidirectional relationship that significantly impacts quality of life (14, 15). Quality of life declines further with advancing age and the presence of chronic diseases (16). Oral diseases such as dental caries, periodontal disease, and tooth loss are more prevalent among those with SMI compared to the general population, contributing to broader health disparities (17). This highlights the urgent need for integrated care approaches that address both mental and oral health to improve overall well-being and reduce the life expectancy gap experienced by individuals with severe mental illnesses.

Despite growing recognition of the bidirectional relationship between mental and oral health, a critical gap persists in understanding the lived experiences and specific oral healthcare needs of individuals with SMI. This qualitative meta-synthesis aims to explore and synthesize existing research to delineate the multifaceted barriers hindering access to dental care for this vulnerable population and identify underlying systemic challenges. By integrating diverse perspectives, the study seeks to inform the development of targeted interventions aimed at improving oral healthcare challenges and reducing disparities. Ultimately, this work highlights the imperative for holistic care models that concurrently address both mental and oral health needs to enhance overall well-being in individuals with SMI.

## Methods

### Inclusion and exclusion criteria

This review included qualitative research on adolescents and adults (15+) diagnosed with SMI, such as schizophrenia, bipolar disorder, major depression, and psychotic disorders. Studies were eligible if at least 75% of participants met the SMI criteria or if separate results were provided for this group. Research conducted in any setting, including community-based environments, psychiatric hospitals, or residential care facilities, was considered. All study designs were accepted if the articles focused on oral health care-related challenges, such as oral hygiene behaviors, access to dental care, barriers, facilitators, and impact on quality of life. Interventions aimed at improving oral health or addressing healthcare barriers were included. Only peer-reviewed, English-language studies were considered, ensuring high-quality evidence. Studies were excluded if they involved individuals without an SMI diagnosis or those under legal guardianship, lacked a focus on oral health interventions, or were non-peer-reviewed publications, editorials, conference abstracts, or non-English articles. These criteria helped maintain a clear and relevant synthesis of qualitative evidence on oral health challenges and interventions for individuals with SMI.

### Search strategy

A comprehensive literature search was conducted across multiple databases, including PubMed, Web of Science, Scopus, and Google Scholar. The search strategy utilized Boolean operators and truncation to identify relevant studies on “serious mental illness” and “oral health.” Search terms included various SMI diagnoses such as “schizophrenia,” “bipolar disorder,” “major depression,” and “psychotic disorders,” combined with “oral health”-related terms like “dental care,” “oral hygiene,” and “oral health-related quality of life (OHRQoL).” Additional terms such as “focus groups,” “interviews,” “thematic analysis,” “content analysis,” “grounded theory,” and “phenomenology” were incorporated to ensure the inclusion of qualitative research. This search strategy was designed to capture a broad yet specific range of studies examining the intersection of oral health or oral diseases and mental illness through qualitative methodologies. We limited our search to articles published between January 2010 and January 2025. The study is registered under PROSPERO (CRD42024516535).

### Severe mental health

Severe mental illness (SMI) is defined as a subset of mental disorders that are particularly debilitating and significantly impair an individual's ability to function in daily life. It typically includes schizophrenia and other psychotic disorders, bipolar disorder, and major depression with psychotic features. The key characteristics of SMI include:

1. Severe functional impairment in major life activities
2. Persistent and long-term nature of the illness
3. Often includes psychosis as a component
4. Higher risk for anosognosia (lack of illness insight)
5. Increased vulnerability to negative outcomes such as homelessness, hospitalization, and criminal justice involvement

### Poor oral health

Oral health encompasses the condition of the mouth, teeth, and orofacial structures, impacting physical functions like eating and speaking, and mental and emotional well-being. Poor oral health commonly includes dental caries (tooth decay), periodontal (gum) disease, tooth loss, and oral cancers. According to the FDI World Dental Federation (18), oral health is defined as:

“Oral health is multifaceted and includes the ability to speak, smile, smell, taste, touch, chew, swallow, and convey a range of emotions through facial expressions with confidence and without pain, discomfort, and disease of the craniofacial complex.” Oral health care is considered a fundamental component of overall health and well-being, reflecting physiological, social, and psychological attributes essential to quality of life (18, 19). However, poor oral health not only causes pain, discomfort, and disfigurement but is also linked to broader health issues, including cardiovascular disease, diabetes complications, respiratory infections, and adverse pregnancy outcomes.

### Data extraction and analysis

For this qualitative meta-synthesis, we conducted a comprehensive data extraction and analysis. Data from the included studies were systematically organized into a standardized Excel template to ensure consistency and accuracy. We categorized extracted data into key study details, including author, publication year, country, and participant demographics such as age, gender, and SMI diagnosis. Methodological aspects were also recorded, encompassing data collection methods (e.g., interviews, focus groups) and analysis approaches (e.g., thematic analysis, grounded theory). To capture the core findings, we extracted key themes related to barriers, facilitators, experiences, and the overall impact of oral health on individuals with SMI. This included both direct participant quotes and paraphrased findings. Additionally, a critical appraisal of each study was documented to assess quality and reliability.

Manual coding was performed on the selected articles to identify patterns and recurring themes. Finally, we conducted a synthesis by comparing findings across studies to ensure consistency and robustness. A visual mapping approach was employed to illustrate the relationship between SMI symptoms, barriers to oral healthcare, and the subsequent deterioration of mental health, providing a clearer understanding of the interconnected challenges faced by this population.

### Quality and risk of bias assessment

Using the CASP (Critical Appraisal Skills Programme) Qualitative Checklist in this meta-synthesis ensured a rigorous assessment of study quality and potential biases (20). This tool evaluated qualitative research validity through ten key questions, covering aspects such as the appropriateness of study design, recruitment strategy alignment with research objectives, reflexivity, and ethical considerations. By systematically applying these criteria, the study maintained a high standard of evidence selection.

Studies with critical flaws, such as ethical violations or a lack of theoretical framework, were excluded to enhance reliability. The classification system—high quality (≥80% criteria met), moderate quality (50%–79%), and low quality (<50%)—helped to differentiate the strength of the included studies. This tiered approach ensured that findings were derived from well-conducted research while acknowledging variations in study rigor. All assessment decisions were documented using a PRISMA flow diagram for transparency and reproducibility, with assessment tables appended to the meta-synthesis. This methodological clarity strengthened the validity of the synthesis by allowing for independent verification and replication. By adopting a structured appraisal process, the study ensured a comprehensive and credible analysis of qualitative evidence on oral health challenges among individuals with serious mental illness.

## Results

### Identification of published and relevant literature

**Figure 1:**
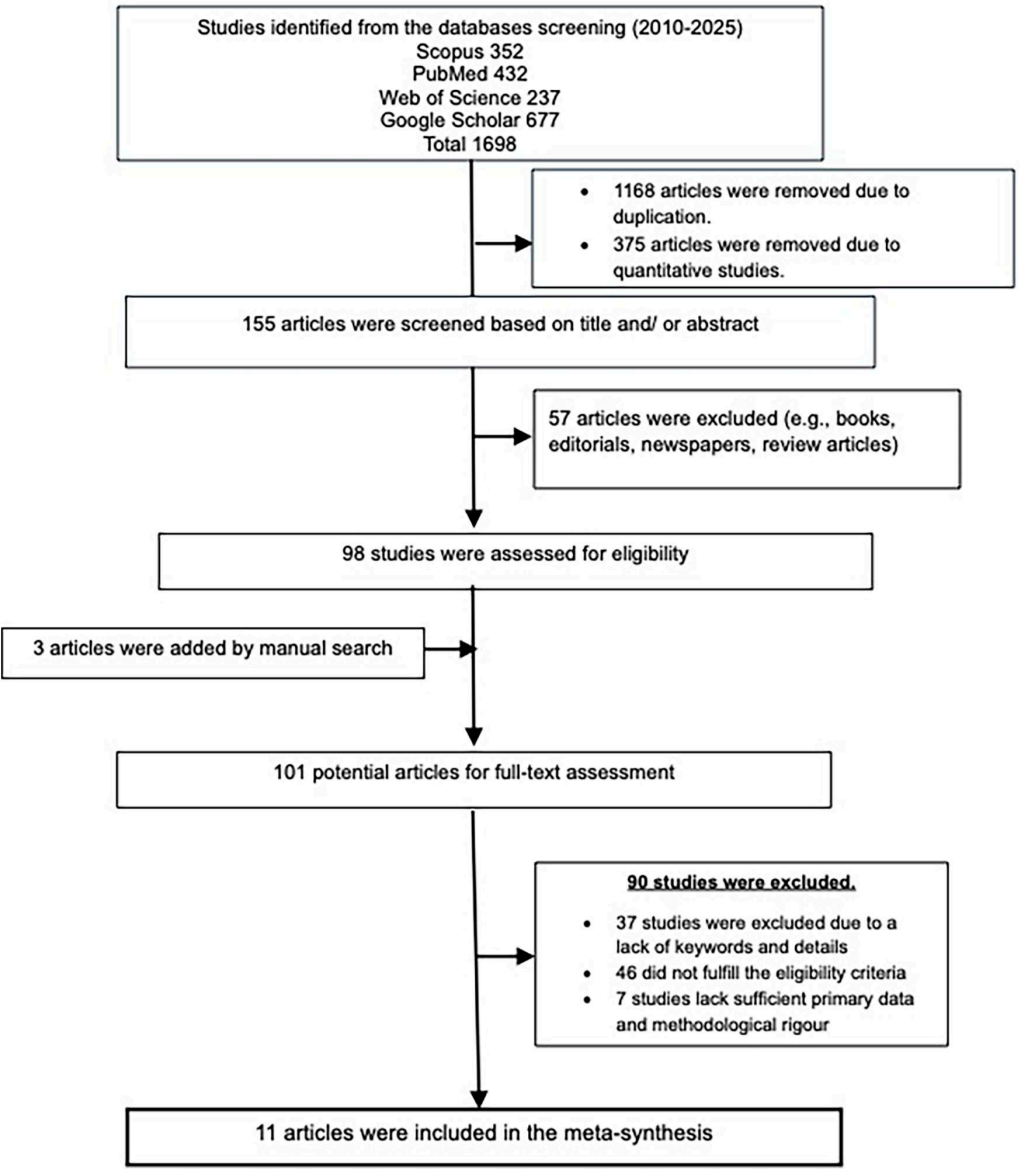
Prisma checklist for article selection.

The systematic search initially identified 1,698 records, which were reduced to 155 after removing duplicates and irrelevant entries (Figure 1). The other 57 records were excluded as they comprised books, editorials, and review articles. Three articles were added manually. A total of 101 articles underwent full-text review to assess sample characteristics, qualitative methodologies, and their focus on exploring nature experiences. Of these, 18 were identified as highly relevant and further assessed using the CASP Qualitative Checklist. Seven studies were excluded due to insufficient primary data or methodological limitations, as they lacked insights grounded in participants’ perspectives. Ultimately, 11 studies were selected for synthesis, and the CASP evaluations of the articles are given in https://osf.io/fu4m6/.

### Characteristics of included documents

Table 1 provides an overview of the included studies published between 2010 and 2024, with the majority (8 out of 11) appearing after 2020 (21–30). Sample sizes for in-depth and key informant interviews ranged from 8 to 65 participants. The studies were conducted across eight countries: the United States (n = 2), France (*n* = 2), the United Kingdom (*n* = 2), the Netherlands (*n* = 1), Jordan (*n* = 1), Australia (*n* = 1), Sweden (*n* = 1), and Norway (*n* = 1). Notably, a study by Couatarmanach et al. (27) interviewed dentists as key informants to gain insight into dentists’ experiences with the lived realities of their SMI patients, particularly those affected by specific oral health challenges.

**Table 1:** Characteristics of the included articles for meta-synthesis.

Authors	Publication year	Country	Study methods and materials	Participants’ criteria
Kalf-Scholte et al.	2024	Netherlands	Using a qualitative, descriptive approach, the study conducted focus groups and interviews with 12 adolescents, 9 counselors, and 6 oral health professionals (OHPs). Thematic analysis identified factors influencing adolescents’ knowledge, attitudes, planning, and execution of oral health behaviors, linking these to oral care stakeholders.	This study focused on adolescents with mild to borderline intellectual disabilities living in residential care in the Netherlands. Adolescents in such facilities live in group homes, supported and supervised by counsellors.
Mishu et al.	2022	UK	The qualitative study used convenience sampling on recruited service users aged over 18 years, living in the UK, with a self-reported diagnosis of serious mental illness (SMI), including schizophrenia, schizoaffective disorder, or bipolar disorder. In-depth interviews were conducted using either one-to-one or dyadic interview techniques.	People over the age of 18 years. Living in the UK. Having had self-reported diagnosis of SMI (schizophrenia, schizoaffective disorder or bipolar disorder).
Bjørkvik et al.	2022	Norway	The study employed a flexible qualitative design with semi-structured face-to-face interviews conducted twice with 51 individuals with serious mental illness (SMI)—once before dental treatment and once after. Thematic analysis was used to analyze the data.	Participants mean age was 42.6 and maximum age was 78 years. Mental health disorders (anxiety, mood disorder, bipolar disorder). Duration of illness was considered more than 5 years.
Hansen et al.	2021	UK	The focus group comprised disabled patients (Parkinson's disease, autism, dementia, schizophrenia). Individuals with disabilities encounter specific challenges that hinder their access to proper care, and a thematic analysis was conducted.	Disabled people (physically, mentally, intellectually or sensory in nature)
Wright et al.	2021	USA	The study involved semi-structured interviews with 65 participants at an academic medical campus in rural North Carolina.	65 individuals with mental illness, incorporating perspectives from patients, psychiatrists, and dentists.
Denis et al.	2021	France	The qualitative study took place in France's Côte d'Or department and involved semi-structured interviews with 20 people with schizophrenia (12 males, 8 females) with a mean age of 45.8 years.	20 Schizophrenic (PWS) patients, with a median age between 45.8 (±9.5).
Hassona et al.	2021	Jordan	The qualitative study includes interviews on the oral health care experiences of individuals with intellectual disability (ID) and their families in Jordan.	The study involved semi-structured interviews with 26 parents of individuals with ID (autism, down syndrome, ADHD), with an average age of 46.4 years for parents and 17.9 years for individuals with ID.
Couatarmanach et al.	2020	France	The qualitative study explored dentists’ perspectives on barriers to providing oral health care in French psychiatric hospitals with on-site dental clinics. The study involved semi-structured interviews with eight dentists working in psychiatric facilities.	Eight dentists working in psychiatric facilities.
Ho et al.	2017	Australia	The study involved focus groups and semi-structured interviews with 12 participants aged 18 and over who had been diagnosed with a mental illness.	12 people living with serious mental illness in the Victorian community.
McKibbin et al.	2014	USA	This study explored the barriers and facilitators to oral health care among 25 adult community mental health outpatients with serious mental illness (SMI) using qualitative methods. Participants took part in 30–60 min semi-structured interviews, which were recorded and transcribed.	Adult people who have serious mental health illness (SMI). 25 adult patients. People who are nor financially solvent, less educated and visited a mental health center in the Rocky Mountain West.
Persson et al.	2010	Sweden	Content analysis of 67 informal interviews with ten participants from two community-based urban housing projects identified five categories: shame related to poor dental health, dental care history, self-care experiences, management of oral health problems, and staff support experiences.	People with serious mental illness (SMI) and were taking psychiatric medications experience oral health problems (especially dry mouth).

### Synthesis

The studies explored various experiences, with the initial coding process providing a broad understanding of the material and highlighting key concepts. Notably, different researchers used varied interpretative language when discussing similar ideas. This meta-synthesis prioritized identifying barriers to oral health care during the coding process while also considering all extracted findings, including individual authors’ interpretations.

To structure the analysis, codes were grouped into logical clusters, leading to the identification of seven core themes: barriers to oral health care, the impact of medications, oral health-related quality of life, oral health status, facilitators for improved care, dietary habits and substance use, and stigma and social isolation. These shared and interconnected meanings are investigated under the core theme of barriers to oral health care.

Table 2 presents the thematic findings, citing source documents, followed by a detailed summary of each core theme with references to descriptive themes. However, as the primary objective of this study is to explore barriers to oral health care, the discussion focuses exclusively on the subthemes under this core theme.

**Table 2:** Summary of thematic findings.

Core theme	Description	Supporting articles
Barriers to oral health care	- High costs and financial constraints in seeking oral healthcare. - Fear of dental procedures, lack of transportation, and stigma associated with mental illness. - Lack of education, awareness, and systemic support for addressing oral health - Missing dental appointment and patients reluctancy of accepting further appointments. - Limited access to dental services and under-utilization of care. - Cognitive impairments and lack of motivation for self-care.	[21–31]
Impact of medications	- Side effects like xerostomia, bruxism, and tardive dyskinesia caused by psychotropic medications. - Medication-induced changes in oral health behaviors and increased risk of caries and periodontal disease.	21,22,23,25,31
Oral health-related quality of life (OHRQoL)	- Poor oral health contributes to social isolation, low self-esteem, and reduced quality of life. - Individuals with SMI report significantly higher OHRQoL impact scores compared to the general population.	21,23,25
Oral health status	- Higher prevalence of dental caries, periodontal disease, tooth loss, and oral mucosal lesions compared to the general population. - Poor oral hygiene practices due to neglect and lack of awareness.	21,24,25,26
Facilitators for improved care	- Integrated mental health and oral health services. - Education and training for providers on managing patients with SMI. - Community support programs and tailored interventions to improve oral hygiene practices.	24,25,27
Dietary habits and substance use	- High consumption of cariogenic foods/drinks, substance misuse (tobacco, alcohol), and poor nutrition exacerbate oral health issues. - Sensory sensitivities in some disorders (e.g., ASD) lead to dietary challenges affecting oral health.	21,22
Stigma and social isolation	- Stigma from dental professionals and society discourages individuals with SMI from seeking care. - Poor oral aesthetics lead to low self-confidence, withdrawal, and reduced social participation.	21,24,25,31

#### High costs and financial constraints on oral health care

The studies highlighted significant barriers to oral healthcare access for individuals with mental illness, with cost being a central issue. Patients, psychiatrists, and dentists were interviewed across multiple studies, revealing that financial constraints often prevent individuals from seeking preventative or follow-up dental care. Many patients only visit dentists during emergencies due to high treatment costs, lack of affordable insurance, and limited availability of public dentists accepting health coverage (23, 24). Low income, unemployment, and higher living costs exacerbate these challenges, with indirect expenses like transportation and wage loss further limiting access (3, 21, 22, 29).

Participants frequently cited difficulty finding dentists who accept public insurance, particularly in remote areas, and expressed concerns about unpredictable eligibility for free care (3, 29). Fear of high costs and uncertainty about payment also deterred individuals from seeking timely treatment, with some choosing to endure pain rather than incur expenses (28). Patients emphasized the need for transparent information about fees, payment plans, and discounts to alleviate financial anxieties and improve access to care (30). These findings underscore the need for systemic changes to address economic barriers and ensure equitable oral healthcare for individuals with mental illness. A participant from a study stated the reason for not getting continued dental care was fear of not having enough money to pay for services.

“Haven't been to the dentist for a few years because of financial reasons.” [Article from Ho et al., 2017 (28)]

#### Fear of dental procedures and stigma associated with mental illness

Dental anxiety and fear are significant barriers to care, often heightened by psychological stress and past negative experiences (22, 24, 29). Low confidence about oral health can reduce social interaction and worsen mental health, leading to isolation (30). The stigma surrounding mental illness and intellectual disabilities further discourages individuals and families from seeking dental care, with some avoiding public spaces due to societal judgement (26, 28). Additionally, the invasive nature of dental procedures and lack of trust in dental professionals contribute to reluctance to seek treatment (22, 28, 30). Moreover, parents of children with intellectual disabilities reported societal stigma, which limits their ability to seek care and participate in social life (23). Furthermore, parents of disabled children with exacerbated dental disease reported late-stage presentations due to pain or swelling. They often received reactive care without preventive advice, leading to a cycle of medication and referrals (23). One of the study participants described the scenario highlighted in the research:

“I experience shame more than anxiety at the dentist and expect to be ridiculed or criticized. I have been bullied before, and I always expect it to happen again. Trust is difficult.” [Article from Bjørkvik et al., 2021 (3)]

Another article stated the same scenario from a patient:

“I have anxiety attacks at the dentist’s office… I do not take things easily and feel stressed when I have dental pain… I am afraid to go to the dentist… I have difficulties in managing my visit to the dentist.” [Article from Denis et al., 2021 (25)]

#### Lack of education, awareness, and systemic support for addressing oral health

Both dentists and psychiatrists identified gaps in their education, with dentists lacking training on the link between oral and mental health and psychiatrists requiring additional training on the importance of oral health screening (24). Patients’ lack of dental insurance and awareness about the importance of oral health further deterred them from seeking care (24). Many delayed treatments until emergencies arose, opting for extractions over prolonged procedures (30). Poor dietary habits and stress-related behaviours, like excessive snacking, negatively impact oral health (25). While some patients with better oral health literacy sought regular care, others delayed treatment until experiencing severe pain or swelling, often receiving reactive rather than preventive care (26, 29). Communication barriers, including complex dental terminology, further hindered understanding and effective care (28). One dentist from a hospital stated that

“There are 30%–40% of the patients who refuse dental care; they say, ‘No, I’m OK, like that, don’t touch my mouth”, and the nurse called us to say that he’s not going to come to the appointment, and we’re not going to force him to come.” [Article from Wright et al, 2021 (24)]

These findings highlight the need for improved education, preventive strategies, and clearer communication in oral healthcare for both patients and doctors.

#### Missing dental appointments and patient experiences

Dental providers face challenges in managing high caseloads and often refer patients who miss appointments, while patients struggle to find accessible and timely care due to overburdened services (22, 23). Some patients with severe mental illness value their independence and perceive reminders or offers of assistance as patronizing, preferring to manage their oral care without external support (30). Cognitive impairments affect oral hygiene routines. One of the patients from a study highlighted,

“On days when I feel hopeless and think of ending life, I forget both to brush my teeth and to go to the dental clinic.” [Article from Bjørkvik et al., 2021 (3)]

These issues highlight the need for better resource allocation and patient-centered approaches in dental care, especially for people with mental illness.

#### Limited access to dental services and underutilization of care

Limited access to dental services, whether through the National Health Service or private providers, discourages health-seeking behaviours across all demographics, with long waiting lists and high demand being significant barriers (22, 23). Balancing adequate care with efficient use of public funds remains a challenge, requiring careful planning by healthcare commissioners. The social model of disability is highlighted as a better framework for understanding the barriers faced by individuals with disabilities in accessing healthcare (23).

## Overview of findings

**Figure 2:**
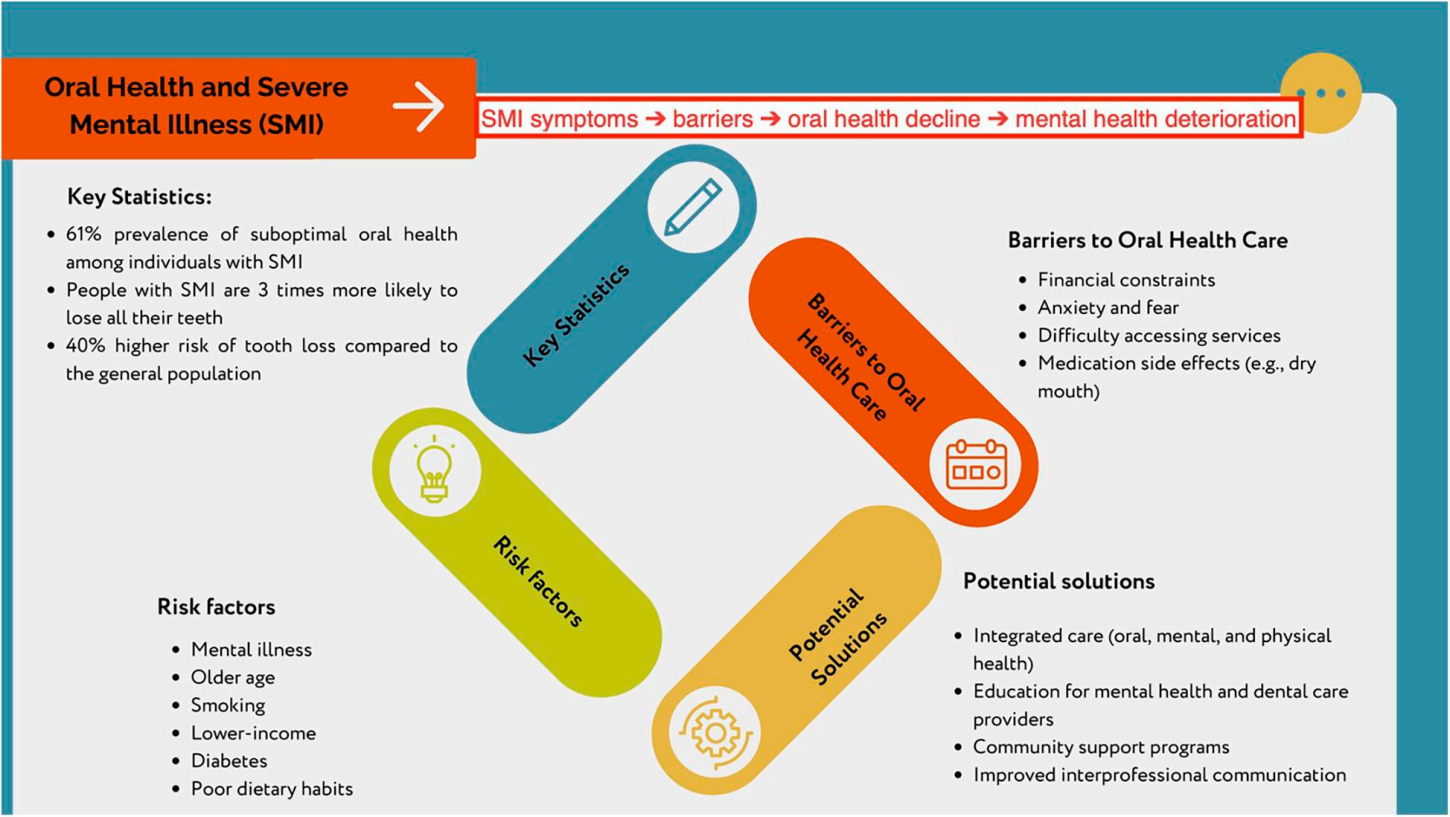
Summary of findings from the meta-synthesis.

Figure 2 presents a four-step cycle, illustrating the significant oral health challenges faced by individuals with severe mental illness. Key statistics show a 61% prevalence of suboptimal oral health, a threefold likelihood of losing all teeth, and a 40% higher risk of tooth loss than the general population (3). Risk factors include mental illness, old age, smoking, lower income, diabetes, and unhealthy dietary habits. Barriers to care include financial constraints, anxiety, difficulty accessing services, and medication side effects, for example, xerostomia. Potential solutions proposed are integrated care, improved provider education, community support programs, and enhanced interprofessional communication.

## Discussion

The meta-synthesis reveals systemic barriers to oral health care for individuals with serious mental illness (SMI), with financial constraints, fear/stigma, and fragmented care systems emerging as critical challenges. These findings align with existing literature while highlighting the unique challenges faced by this population. Below, we contextualize these barriers using evidence from the reviewed studies.

High costs and limited insurance coverage were consistently cited as prohibitive factors, forcing many individuals with SMI to delay or avoid dental care until emergencies arise. For example, five studies from the meta-synthesis found that participants avoided dental visits due to unaffordable fees. In contrast, another study noted that only 40% of psychiatrists screened patients for oral health due to perceived irrelevance in mental health care. This aligns with broader findings that individuals with SMI have a 60% higher odds of tooth loss compared to their peers without mental health issues (31). Another study found that perceived poor mental health correlates with a 1.90% increase in the likelihood of complete tooth loss (32). Financial constraints are a major barrier to accessing dental care for individuals with SMI, particularly in low- and middle-income countries (LMICs) (33). The lack of public dental services accepting subsidized insurance exacerbates inequities, particularly in rural or underserved areas.

Dental anxiety, compounded by societal stigma toward mental illness, creates a cycle of avoidance and worsening oral health. The synthesis found individuals avoid care due to fear of judgement, and there's a relationship between poor oral health, shame, and social withdrawal. Mental health stigma also discourages families of individuals with intellectual disabilities from seeking care, as reported. Many individuals experience dental anxiety due to fear of pain and negative past experiences, leading to avoidance of dental care (34). This anxiety is influenced by intrapersonal, interpersonal, and societal factors, including personal traits, past encounters, and societal stigma toward mental illness, which can deter individuals from seeking help and worsen their oral health over time (34, 35). Notably, the invasive nature of dental procedures and distrust in providers further heighten anxiety, particularly among those with psychiatric conditions.

Dental and mental health professionals reported insufficient training to address the interplay between oral health and SMI. The meta-synthesis explored that dentists cited poor communication with psychiatric staff while psychiatrists lacked awareness of oral health screening protocols. The finding is consistent with other studies (36, 37). For example, many dental professionals lack formal training in mental health, leading to communication challenges and treatment hesitancy (36). A survey of dental students highlighted fear and discomfort in treating patients with SMI, underscoring the need for improved education (37). Additionally, medication side effects and dietary habits contribute to poor oral hygiene, further worsening mental health.

Access to dental care for individuals with severe mental illness (SMI) is hindered by systemic and socioeconomic barriers, exacerbating oral health disparities. Long waiting lists and overburdened public health systems delay treatment, often leading to the progression of untreated dental conditions. Quantitative studies also evaluated these and found similar results (38, 39). Geographic disparities further restrict access, particularly in rural or underserved areas where specialized dental services are scarce (40). For those with SMI, these financial obstacles compound existing challenges, making regular dental visits difficult to maintain (31, 32).

The synthesis noted that patients with SMI often miss dental appointments and may not recognize the severity of their oral health issues due to various barriers. Additionally, cognitive impairments associated with SMI, such as apathy, executive dysfunction, and disorganized thinking, further reduce adherence to oral hygiene routines. Individuals may struggle with daily brushing and flossing, miss dental appointments, or fail to recognize the severity of their oral health issues. One study mentioned that individuals with SMI face challenges such as managing complex life circumstances, psychological and physical difficulties, and a lack of motivation or awareness regarding oral health (41). This neglect creates a cycle in which poor oral health exacerbates physical discomfort and social withdrawal, ultimately worsening overall well-being and mental health outcomes. Accessibility and availability of dental services are also significant issues, with many patients experiencing lower rates of dental service utilization compared to the general population (42).

The relevance of these findings to low- and middle-income countries (LMICs) warrants careful consideration, as differences in healthcare infrastructure, socioeconomic conditions, and cultural contexts significantly shape outcomes. In LMICs, the already high prevalence of oral health problems such as dental caries and periodontal disease among individuals with SMI is likely exacerbated by limited access to preventive and curative dental services (particularly in rural areas where dentists are scarce), a higher burden of malnutrition and systemic illnesses like diabetes, and pervasive stigma surrounding mental illness (43–47). These challenges are compounded by fragmented health systems with minimal integration between mental health and dental services, a shortage of dental professionals trained to care for individuals with SMI, and substantial financial barriers, including the absence of universal health coverage and heavy reliance on out-of-pocket payments.

### Strengths and limitations of the study

This review synthesizes qualitative research to provide a comprehensive view of oral health challenges in individuals with severe mental illness (SMI). It captures lived experiences, highlighting key themes such as barriers to care, medication side effects, stigma, and systemic challenges. Common issues include financial constraints, lack of mental health training among dental professionals, and medication-induced xerostomia. Stigma and social isolation further hinder access to care. While enhancing the generalizability of insights, the study also suggests solutions like integrated care models and interdisciplinary collaboration. However, the following limitations should be considered in broader applications of this meta-synthesis.

This meta-synthesis has several limitations that should be considered when interpreting its findings. First, the study is based on participants with serious mental illness (SMI) in the Global North, limiting the generalizability of results to other clinical or geographic populations. While efforts were made to conduct comprehensive sampling, the scarcity of relevant literature resulted in a small sample size (*n* = 11). Another challenge lies in the heterogeneity of study designs, populations, and interventions, making direct comparisons difficult. The included studies also exhibited methodological limitations, such as researcher reflexivity, which may influence confirmability and introduce bias in recruitment and research procedures. The synthesis prioritized participants’ raw data (direct quotes) to enhance credibility, ensuring interpretations remained close to their lived experiences. Additionally, detailed documentation of search procedures and quality appraisals was maintained to uphold rigour and transparency.

A further limitation is the potential for researcher bias in interpreting and synthesizing qualitative data, which can influence the conclusions. Lastly, the lack of longitudinal studies prevents an assessment of long-term changes in oral health behaviours and the sustained impact of interventions over time. Future research should address these gaps by incorporating diverse populations, minimizing methodological inconsistencies, and conducting long-term follow-up studies to evaluate intervention effectiveness.

## Conclusion

This meta-synthesis highlights the profound and pervasive burden of poor oral health among individuals with SMI, marked by significantly elevated rates of dental caries, tooth loss and periodontal disease. Key drivers—including suboptimal oral hygiene, limited health literacy, the adverse effects of long-term psychotropic medication (notably xerostomia), and formidable barriers to accessing dental care—converge to create a stark health disparity. Financial hardship, inadequate training of dental professionals in mental health, pervasive stigma, and social isolation further compound this inequity. Addressing this crisis demands coordinated action: the integration of mental and oral healthcare through co-located services, robust interdisciplinary collaboration, and the implementation of tailored, accessible interventions is imperative. Future research must prioritise quantitative studies to elucidate causal pathways and long-term impacts, rigorously examining the roles of gender, geography, environmental factors, and comorbid physical conditions. Bridging this divide is not merely a dental concern but an urgent public health imperative, requiring systemic reform to uphold the fundamental health rights of this vulnerable population.

## Data Availability

The original contributions presented in the study are included in the article/Supplementary Material, further inquiries can be directed to the corresponding author.
